# Engineering surgical stitches to prevent bacterial infection

**DOI:** 10.1038/s41598-022-04925-5

**Published:** 2022-01-17

**Authors:** Daniela Vieira, Samuel N. Angel, Yazan Honjol, Maude Masse, Samantha Gruenheid, Edward J. Harvey, Geraldine Merle

**Affiliations:** 1 Experimental Surgery, Faculty of Medicine, McGill University, Montreal, H3A 2B2 Canada; 2 Department of Microbiology, Faculty of Medicine, McGill University, Montreal, H3A 2B2 Canada; 3 Department of Surgery, Faculty of Medicine, McGill University, Montreal, H3A 0C5 Canada; 4 Chemical Engineering Department, Ecole Polytechnique de Montréal, P.O. Box 6079 Station, Montreal, QC H3C 3A7 Canada

**Keywords:** Biomedical engineering, Biomedical materials

## Abstract

Surgical site infections (SSIs) account for a massive economic, physiological, and psychological burden on patients and health care providers. Sutures provide a surface to which bacteria can adhere, proliferate, and promote SSIs. Current methods for fighting SSIs involve the use of sutures coated with common antibiotics (triclosan). Unfortunately, these antibiotics have been rendered ineffective due to the increasing rate of antibiotic resistance. A promising new avenue involves the use of metallic nanoparticles (MNPs). MNPs exhibit low cytotoxicity and a strong propensity for killing bacteria while evading the typical antibiotic resistance mechanisms. In this work, we developed a novel MNPs dip-coating method for PDS-II sutures and explored the capabilities of a variety of MNPs in killing bacteria while retaining the cytocompatibility. Our findings indicated that our technique provided a homogeneous coating for PDS-II sutures, maintaining the strength, structural integrity, and degradability. The MNP coatings possess strong in vitro antibacterial properties against *P aeruginosa* and *S. aureus*—varying the %of dead bacteria from ~ 40% (for MgO NPs) to ~ 90% (for Fe_2_O_3_) compared to ~ 15% for uncoated PDS-II suture, after 7 days. All sutures demonstrated minimal cytotoxicity (cell viability > 70%) reinforcing the movement towards the use MNPs as a viable antibacterial technology.

## Introduction

Surgical site infections (SSI) are common and costly nosocomial infections^1–3^. According to the 2016 update from the American College of Surgeons and Surgical Infection Society, SSIs account for at least 20% of all hospital-acquired infections in the United-States. They are associated with an increased length of stay and up to an 11-fold increase in the risk of mortality^4^. The annual cost of dealing with SSI related complications in the US is estimated between 3.5 and 10 billion American dollars^2^. The huge costs associated with surgical site infections are due to an increased length of stay, more frequent admissions to emergency departments and readmission^2,3^. Surgical site infections have monetary implications but failure to prevent an SSI results in an incredible amount of undue stress, discomfort, and potentially serious and lasting complications for the patient. 77% of the mortality in patients that present with an SSI is associated with the infection itself^1,2^. Due to the severity and ubiquity of surgical site infections, they have become a non-validated metric of hospital performance and a target for quality of care improvement initiatives^2,3,5,6^.

There are a variety of contributing factors that cause SSIs ranging from systems-based practices to specific surgical issues. To address this problem, the Center for Disease Control (CDC) has outlined many of the measures that hospitals must consider to prevent SSIs. The list ranges from homeostatic measures—such as glycemic control, oxygenation and normothermia—to tool-specific techniques such as antibacterial wound closure methods^5,6^. Large systems-based solutions are difficult to tackle but worthwhile. More specific solutions can be innovation within site-specific measures that are more easily implemented. Wound closure methods have been identified as an area that needs improvement^5–7^. Antibiotic coated sutures for wound closure are available. However, current antibiotics used to coat sutures—such as triclosan—show insufficient effectiveness against bacterial resistance^7–14^. Novel suture coatings with demonstrable antibacterial activity may be a promising new tool in the fight against surgical site infections.

The use of metallic nanoparticles (NPs) has become an increasingly popular area of research due to their non-toxic behavior towards mammalian cells and evidence suggesting the lack of bacterial resistance despite widespread use^15,16^. Many metallic NPs have been shown to possess antibacterial activity in their ionic and colloidal forms by disrupting membrane functionality through metal ion release or/and by damaging the membrane through oxidative stress by reactive oxygen species (ROS)^15,17–19^. The use of metallic NPs against SSIs seems to be desirable for lack of immunogenicity^20^, e.g. Ag NP coatings have been applied to surgical sutures due to their well-known antibacterial activity^21–24^, despite issues associated with their degradability, presence and migration inside the body which may have severe systemic consequences following long-term exposure^25–27^. Therefore, scientific efforts have been directed towards biodegradable metals, such as Mg, Zn, Fe-based^28–30^. ZnO NPs have been demonstrated as antibacterial coating^24^; however, only few works involve coated-sutures and use extensive and complex process^31,32^.

As the basis of this study, we hypothesize that metallic nanoparticles coated on surgical sutures can decrease bacterial adherence and eradicate bacterial activity and growth. A novel dip coating method using water, ascorbic acid and non-magnetic stirring has been developed in our group^33^. This technique has shown to be universally applicable to any type of nanoparticles and any type of surface regardless of topography, size and intrinsic properties. Here we modified the surface of the sutures with non-degradable and fully biodegradable metallic nanostructures. The metal/ metal oxide nanoparticles selection (ZnO, TiO_2_, Fe_2_O_3_, Cu, Cu_2_O and MgO) was made based on their relevance as antibacterial agent and/or biodegradability^20^. The studied suture design features complete metallic NP coating followed by a thin layer of silk fibroin (SF) applied to the suture to not only encourage NP adherence, but also to improve skin tissue regeneration and wound healing^34,35^. Once the coated suture has been in contact with the target environment, the silk degrades allowing NPs to diffuse. We aimed to achieve successful suture coating and adherence; and explored the antibacterial and cytotoxic behaviors of metallic NP coated sutures in vitro*.*

## Results

**Figure 1:**
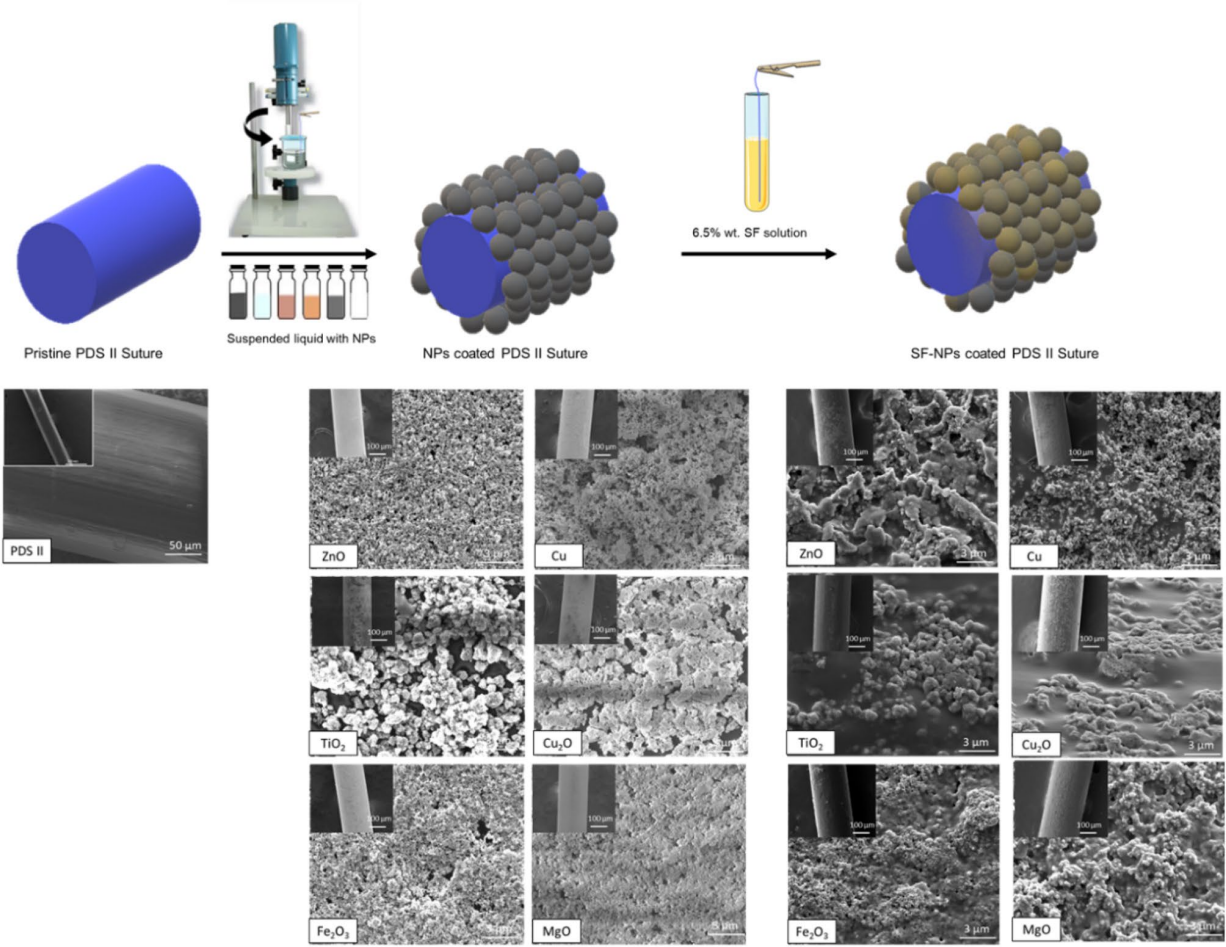
Engineering process of modified PDS II sutures and confirmation of double layer coating via SEM analysis at low (200×) and high (10,000×) magnification.

Figure 1 illustrates the chemical process used to modify the sutures with different metallic NPs. Nanoparticles were suspended in a liquid containing water and ascorbic acid and the mixture was stirred with a vertical rotating rod located off center of the container to facilitate the compression of NPs on the liquid on the surface for approximately 6 h. Transfer of the nanoparticles formed at the surface of the liquid to the sutures was done by dip-coating while the liquid continues to be agitated^36^. After deposition, dried sutures were dip coated in a silk fibroin solution to deposit a thin polymeric layer (Fig. 1). To confirm the deposition of nanoparticle at the surface of the surgical sutures, we performed Scanning Electron Microscopy (SEM). The images obtained before (pristine suture, Fig. 1) and after treatment indicated the presence of substantial amounts of various NPs (Fig. 1). In addition, SEM images of the sutures confirmed that the nanoparticles occupied the whole surface without clustering effect.

**Figure 2:**
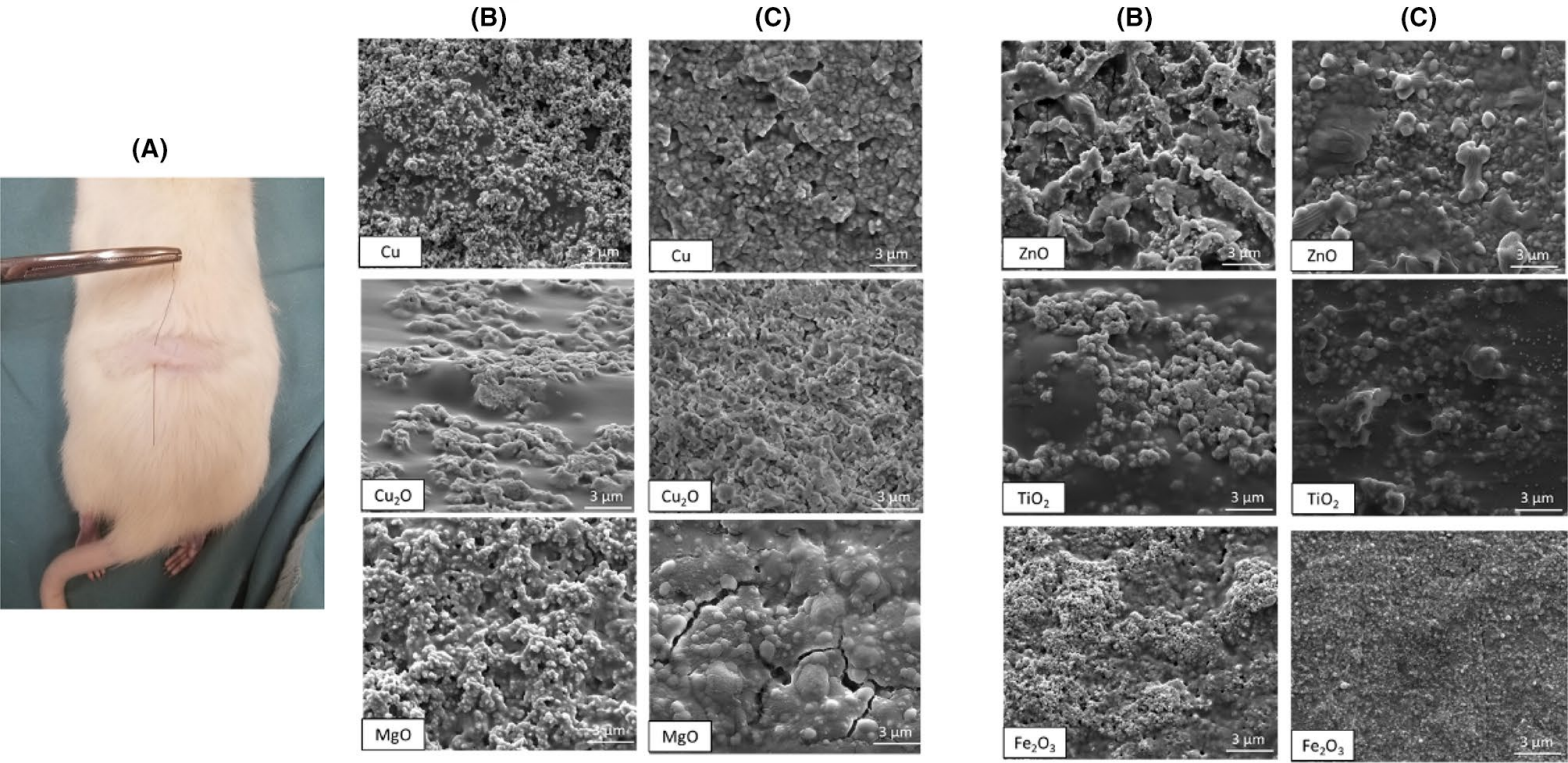
(**A**) The skin model confirmed the retention of NPs by the silk layer on the suture. SEM images showing the nanoparticle coating (**B**) before and (**C**) after pull test results.

In order to secure the attachment of nanoparticles during the suturing, a thin layer of silk-fibroin was deposited on top of the NP coating (Fig. 2). To confirm NP adherence, a skin pull through test was developed and applied to sutures (Fig. 2).

The diameter of the coated sutures was significantly higher (p < 0.05) than the uncoated sutures (Table 1), varying from 224.15 ± 0.81 (for uncoated PDS-II) to 243.34 ± 1.15 (for Cu NPs coating). As expected, the increase was related to the different layers of the NP coatings applied. Since the suture diameter may influence the mechanical properties, we performed a straight-pull tensile test (Table 1). Despite the difference in the diameter, there were no significant differences (p < 0.05) in the stress values across all sutures compared to uncoated PDS-II sutures. However, the strain results varied for the ZnO, Cu, and MgO samples, exhibited a slight decrease in the elasticity when compared to the PDS-II sutures.

**Table 1:** Diameter, stress and strain values for suture conditions.

Suture coating	Diameter (µm)	Strain (%)	Stress (GPa)
Control (PDS II)^(a)^	224.15 ± 0.81	12.7 ± 1.73	1.61 ± 0.07
TiO^a^	236.88 ± 1.77	7.52 ± 0.57	1.72 ± 0.38
ZnO^a^	227.89 ± 0.43	^a^5.31 ± 1.84	1.71 ± 0.06
CuO^a^	241.17 ± 1.59	10.25 ± 2.26	1.86 ± 0.07
Cu^a^	243.34 ± 1.16	^a^5.49 ± 1.28	1.78 ± 0.12
Fe_2_O_3_^a^	233.40 ± 0.84	13.11 ± 1.24	2.02 ± 0.08
MgO^a^	231.88 ± 0.54	^a^6.55 ± 1.14	2.06 ± 0.09

All values represent stress and strain exerted on the suture at the moment of fracture indicating the maximum stress and strain.

^a^ANOVA test p < 0.05—significant difference, n = 5.

**Figure 3:**
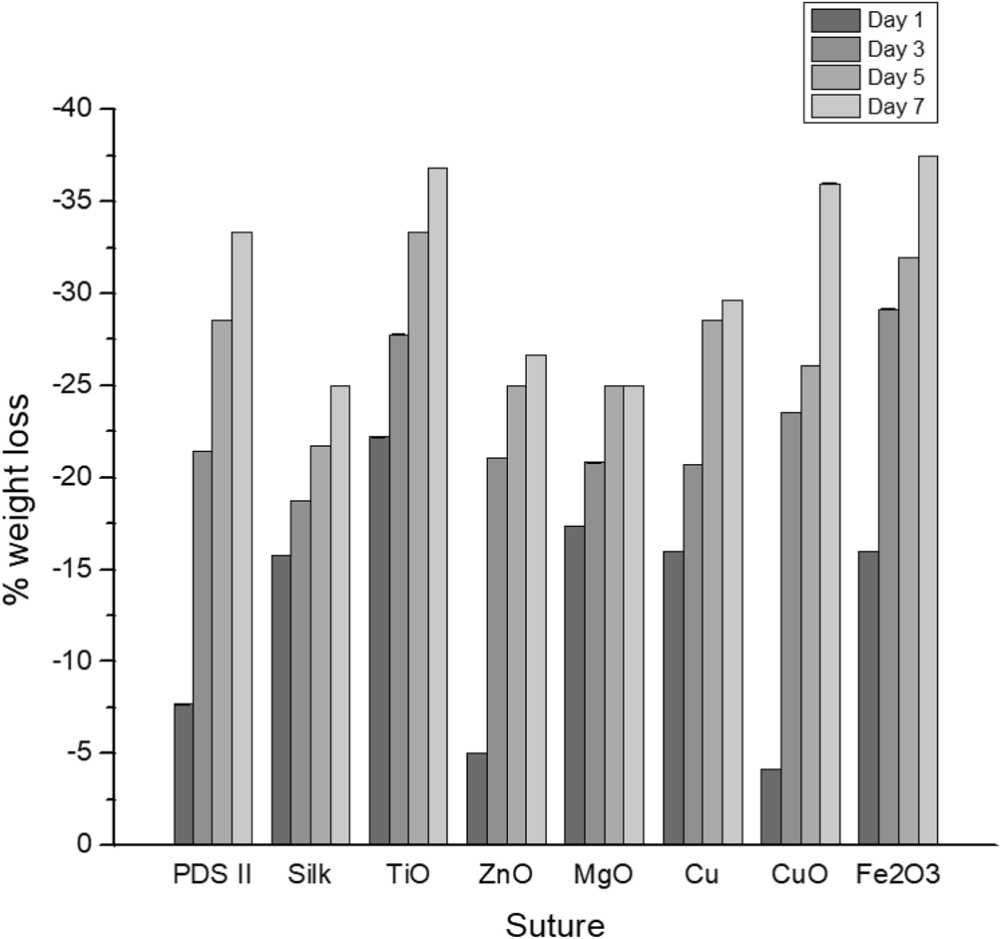
In vitro degradation behavior represented by % weight loss at each timepoint (1, 3, 5, 7 days) after exposure to protease XIV (n = 3). ANOVA and Tukey tests were performed to compare weight differences in coated sutures to weight differences in the uncoated PDS-II samples.

The degradative properties of the suture coatings were tested against the uncoated PDS-II sutures by exposure to protease XIV over 7 days (Fig. 3). After 7 days, all suture conditions including the uncoated PDS II sutures lost around 30% of their initial weight. No significant differences were observed for core suture degradation, showing that the combined layers of metallic NPs and silk-fibroin did not affect the degradation pattern. Statistical significance was determined using ANOVA and Tukey tests.

**Figure 4:**
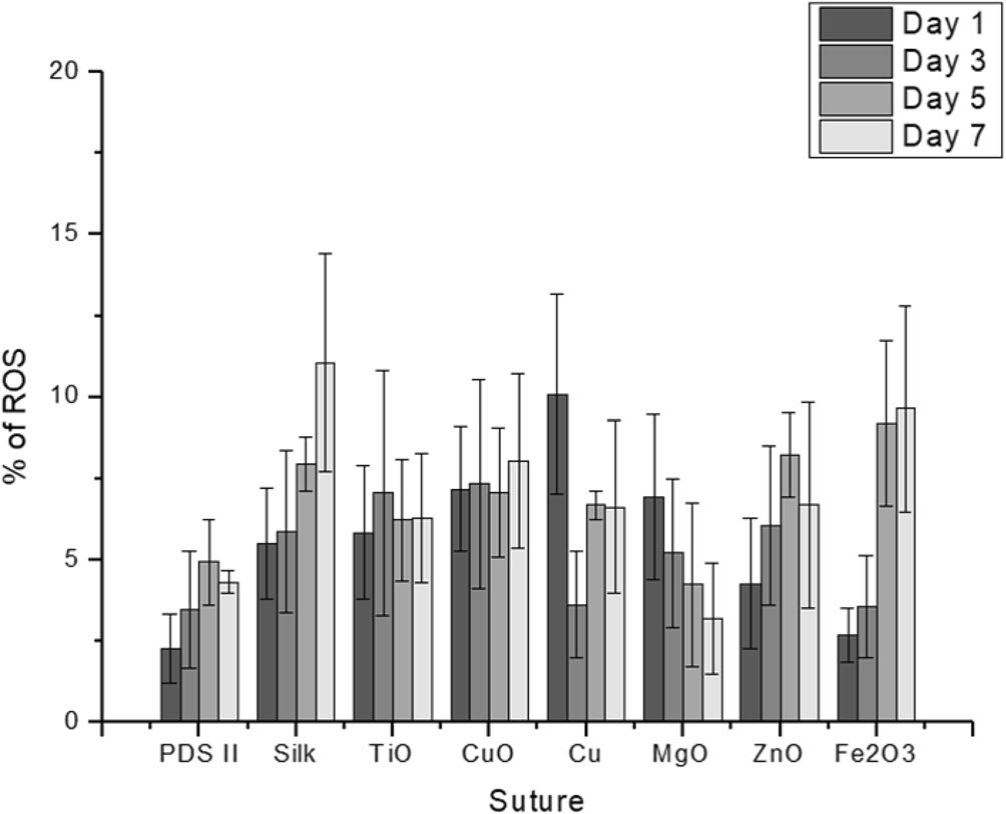
ROS production represented by % of ROS in the aqueous suture testing environment at each timepoint (1, 3, 5, 7 days) (n = 3). ANOVA and Tukey tests were performed with the weight differences in coated against weight differences in the uncoated sutures.

The production of ROS (reactive oxygen species) was examined to evaluate possible effect on the degree of prospective toxicity in the suture microenvironment. The results showed highly variable ROS release across all samples (Fig. 4). The Cu and CuO samples were the only samples to exhibit significantly different results than the control. All other samples and timepoints showed no significant differences.

**Figure 5:**
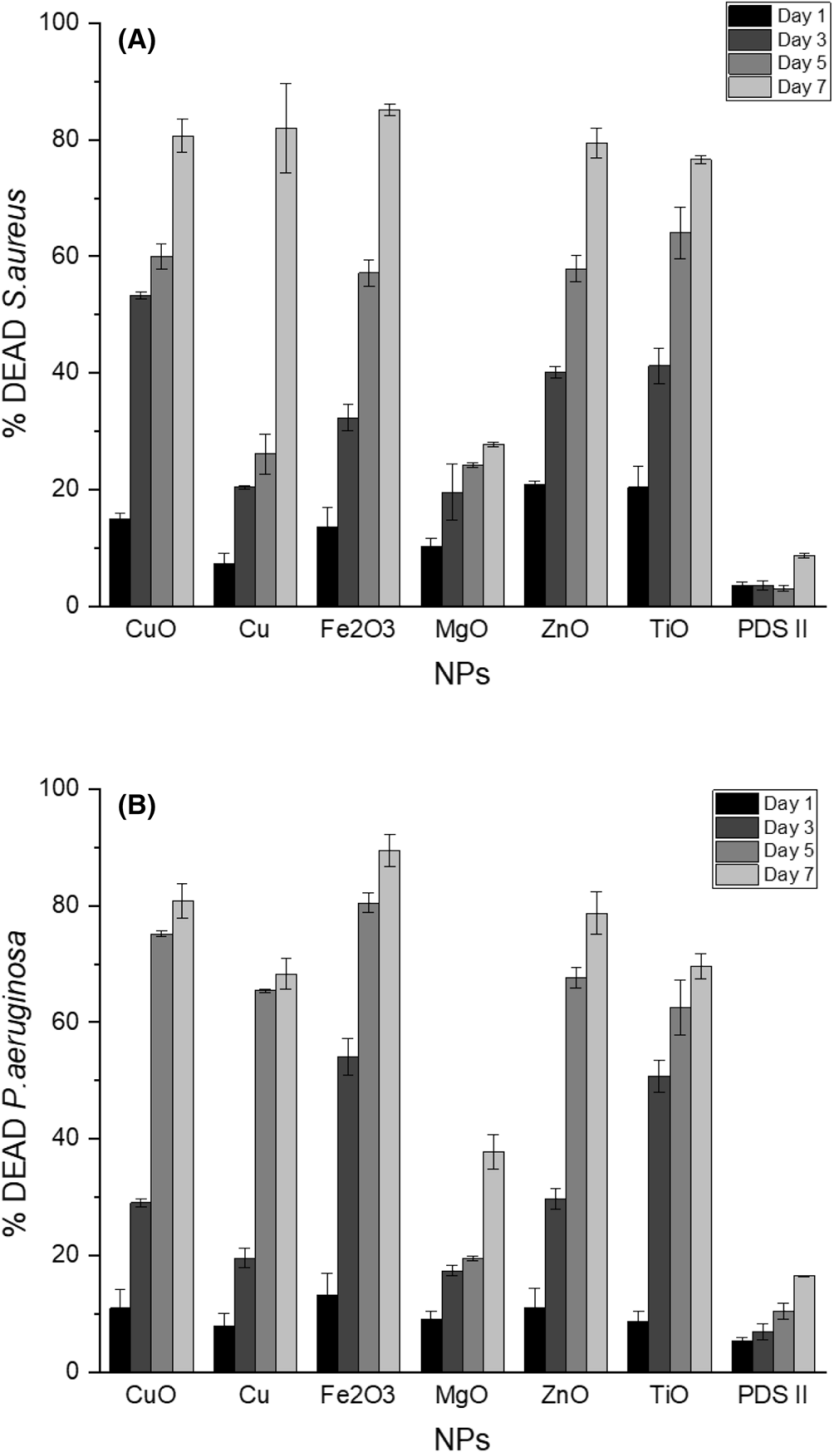
Mean percentage of dead Staphylococcus aureus (**A**) and Pseudomonas aeruginosa (**B**) under different metallic nanoparticle suture coating treatments (n = 12). Four timepoints were observed at 1, 3, 5 and 7 days after exposure to coated suture.

The in-vitro susceptibility of both *Pseudomonas aeruginosa* and *Staphylococcus aureus* to the various metallic nanoparticle coated sutures was assessed (Fig. 5). The results are presented as grouped results from bacterial experiments using *P. aeruginosa* and *S. aureus*, respectively. All data were normalized to the dead negative control by adjusting each dead sample to represent 100% dead bacteria and each experimental condition to be a fraction of the dead control. The PDS II uncoated suture control did not result in bacterial killing greater than 16% in any case. All treated sutures in both bacterial species experiments exhibited progressive bacterial killing behaviors over all time points resulting in at least 68% dead bacteria by day 7—the exception being the MgO coated suture samples which exhibited a maximum bacterial killing at 37.8%. The average amount of dead *S. aureus* in the coated suture samples after day 1, 3 5 and 7 was 14.6%, 32.5%, 46.6% and 71.96% respectively. The average amount of dead *P. aeruginosa* in the coated suture samples after day 1,3, 5 and 7 was 11.2%, 33.5%, 61.8% and 71.8% respectively. All other suture coatings exhibited antibacterial properties resulting in more than 50% dead bacteria by day 5 at the latest and more than 75% dead bacteria on average by day 7. The strongest effect was observed in Fe_2_O_3_ coated sutures—with 85.2% ± 0.9 *P. aeruginosa* dead and 95.4% ± 1.3 *S. aureus* dead. Using GraphPad Prism®, one-way ANOVA tests and Tukey’s tests were performed to assess statistical significance between all treatments at each timepoint with specific emphasis on the comparison between experimental conditions and the uncoated PDS II data. A p-value greater that 0.05 was understood to indicate no significant difference therefore a p-value lesser that 0.05 indicates a statistically significant difference. In the *S. aureus* samples at day 1, only the Cu coated suture samples exhibited a non-significant difference compared to the PDS II samples with p = 0.6451. All experimental *S. aureus* samples at day 1 except the Cu coated sutures exhibited a significant difference in % dead *S. aureus*.

All experimental samples at day 3, 5 and 7 exhibited a statistically significant increase in % dead *S. aureus* when compared to the PDS II samples*.* In the *P. aeruginosa* samples at day 1, only the Zn and Fe_2_O_3_ coated sutures exhibited a significant difference (p = 0.047 and p = 0.002 respectively) when compared to the uncoated PDS II samples. All other *P. aeruginosa* experimental samples at day 1 exhibited a non-significant difference (p > 0.05) when compared to the PDS II samples. All experimental samples at day 3, 5 and 7 exhibited a statistically significant increase in % dead *P. aeruginosa* when compared to the PDS II samples (p < 0.0001).

**Figure 6:**
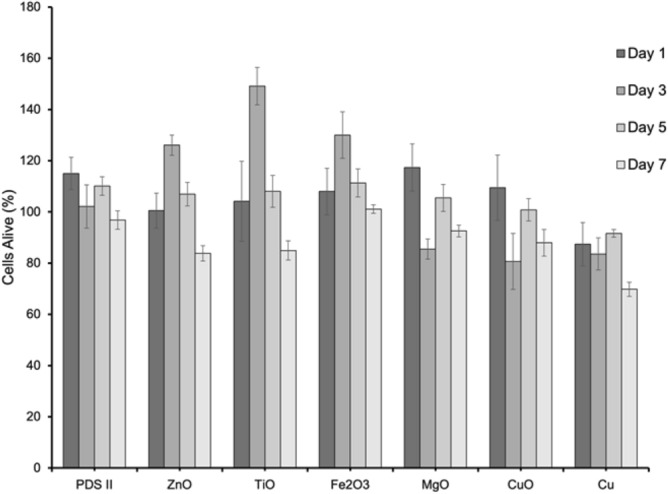
Mean percentage of live CHO cells under different metallic nanoparticle suture coating treatments (n = 12). Four timepoints were observed at 1, 3, 5 and 7 days after exposure to coated suture.

To investigate the cytotoxicity of the various metallic nanoparticle coated sutures, CHO cells were exposed to each treatment exclusively and analyzed at each of four time points outlined (Fig. 6). DNA quantization data were normalized by considering the uncoated samples to express 100% live.

CHO cells therefore all results are expressed in terms of their number of live bacteria relative to the uncoated sample of corresponding timepoint. All mammalian samples exhibited > 70% living bacteria across all timepoints. Using GraphPad Prism®, one-way ANOVA tests and Tukey’s tests were performed to assess statistical significance between treatments at each timepoint to the uncoated samples by which the data were normalized.

## Discussion

Many efforts have been made to prevent and fight SSIs. Techniques used to minimize the incidence of SSIs primarily focus on system-based solutions, such as intensified antiseptic programs and secondly on devices based solutions, such as antibacterial sutures^2,5^. It is understood that surgical sutures act as a viable surface for bacterial adherence resulting in SSI but current antibacterial coatings (i.e. PDS Plus, VicrylPlus®) for sutures are insufficient considering the fact that more than 50% of bacteria are resistant to traditional antibiotics^6,7,9,10,17,37^^.^ Coating strategies aim to combat SSI via two different mechanism: active and passive^38^. Within the active coatings, where compounds are released into the tissue and kill suspended bacteria^38^, peptides^39^ and nanoparticles^22,23,31,32,40^ have emerged as great candidates to fight SSI. Many metallic nanoparticles (NPs)—most notably silver—have been studied against different bacterial strains in several forms including zero-valency, oxide, and molecular complexes conjugates, of different shapes and sizes^16^. Silver nanoparticles is well known in the literature because of its antibacterial effect^17,18,23^; however, little is known about the other metallic nanoparticles. In our work, several metallic NPs (Cu, CuO, TiO, ZnO, Fe_2_O_3_ and MgO) coatings were successfully prepared while maintaining the integrity of the surgical sutures, the antibacterial effect and low toxicity against mammalian cells.

It is important that the underlying function of the suture itself is not changed. Despite the antibacterial property, the ideal suture should not break during the procedure, elongate enough accordingly to the tissue, be compatible and biodegradable^39,41^. The dip-coating method applied in this work is a simple, a non-toxic, and an effective method to coat different substrates^33,36^, providing a homogeneous layer of nanoparticles on the surface of the sutures observed by SEM (Fig. 1). The coating retained the metallic NPs and did not modify the tensile strength for the coatings applied (Table 1). Indeed, the suture bulk material has usually a greater impact on the failure stress than the suture diameter. As an example, the failure stress varied from 0.28GPa for polyglytone sutures to 1.4GPa for polyglactin sutures^41^. In our work, we noted a slightly decrease in elasticity for Cu-, MgO- and ZnO- coated sutures, which may affect in the application for specific tissues where elongation is essential^41^. All suture samples exhibited similar degradation patterns indicating that the coatings do not compromise the longevity of the suture when applied.

The different coatings exhibited minimal cytotoxicity and progressive antibacterial capabilities. The two most common pathogens associated with SSI, *S. aureus* and *P. aeruginosa*^32^, were used to assess the antibacterial properties of our NPs coatings. The Fe_2_O_3_ coating showed the best ability to kill Gram- positive (85.20% ± 0.99) and Gram-negative (89.45 ± 1.32) bacteria (at day 7), followed by Cu, CuO, ZnO, TiO and MgO. The low efficiency of MgO coating could be related to the particle size or agglomeration. It has been noted that large size of MgO particles and/or large area of agglomeration inhibits the interaction with bacteria decreasing, considerable, the antibacterial effect^42^. We strongly believe that the antibacterial effect is directly related to the release of the metal ions as observed in different works in the literature^22,23,31,32,40^; Although the ROS production influences the membrane rupture^17,43^, we did not observe strong action of ROS in this work. The cytotoxicity against mammalian cells showed minimal negative impact on cell life—the cells remaining at least 70% alive after 7 days of contact to the coated sutures. When compared to the standard antibacterial suture PDS Plus, our study revealed exciting findings with similar bactericide effect of Fe_2_O_3_ nanoparticles and triclosan towards Gram + and − bacteria. This is particularly impressive considering the fact that triclosan exposure leads to cross-resistance to antibiotics (Table 2).

**Table 2:** Comparative antibacterial effect and cytotoxicity of different nanoparticles and the standard triclosan coated suture (PDS PLUS).

Suture	% Dead of Gram + ^a^	% Dead Gram − ^b^	% cells alive	Days of assay	References
PDS II (no triclosan)	8.7 ± 0.3	16.5 ± 0.1	97.5 ± 2.9	7	This work
CuO	80.7 ± 2.8	80.8 ± 2.9	88.5 ± 2.7	7	This work
Cu	81.9 ± 7.6	68.3 ± 2.7	70.2 ± 4.5	7	This work
Fe_2_O_3_	85.2 ± 0.9	89.4 ± 1.3	101.8 ± 2.3	7	This work
MgO	27.8 ± 0.4	37.8 ± 2.8	95.5 ± 2.7	7	This work
ZnO	79.4 ± 2.5	78.7 ± 3.6	85.7 ± 3.6	7	This work
TiO	76.6 ± 0.7	69.6 ± 2.1	85.5 ± 1.6	7	This work
Silver NPs	32.5% ± 0.6	35.5% ± 0.5	72.5 ± 8.5	7	^23^
PDS-PLUS (Triclosan)	99.9%	90%	N/A	17	^44^

^a^Gram-Positive: *S. aureus.*

^b^Gram-Negative: *P. aeruginosa* (this work), *E. Coli* (other works).

This work revealed promising properties of certain metallic nanoparticle suture coatings; however, it is important to recognize that this is only the first step in developing such sutures. It is clear that this suture coating methodology is highly functional and attractive in its simplicity, but further testing needs to be done to establish the suture coatings as a viable tool against SSIs (i.e., in vivo tests, suture coatings’ ability to prevent the onset of bacterial colonization, the effect of the mechanical elongation in different tissues, etc.). Furthermore, this work adds knowledge of the antibacterial behavior for a variety of nanoparticles.

## Conclusion

PDS II sutures were successfully coated by an easy and non-toxic dip-coating method using a variety of metallic nanoparticles, proving to be a promising new avenue of research to fight surgical site infections. Metallic nanoparticles were selected for study as they are a novel antibacterial treatment in SSI prevention and are considered less susceptible to bacterial resistance than classical antibiotics, such as triclosan. Following the assessment of biocompatibility towards CHO mammalian cells, the sutures were tested against *S. aureus* and *P. aeruginosa*. The coatings demonstrated promising behaviors, exhibiting great cytocompatibility as well as fast and strong antibacterial behavior. Among the different nanoparticle coatings, Fe_2_O_3_ layer exhibited the greatest bactericide effect towards Gram- positive (85.20% ± 0.99) and Gram-negative (89.45 ± 1.32) bacteria (at day 7), followed by Cu, CuO, ZnO, TiO and MgO. To further reduce bacterial proliferation and increase bacterial eradication, the effect of Fe_2_O_3_ size and shape could be envisaged. This study confirms the potential and simplicity of using metallic nanoparticle coated sutures in the fight against SSI without the use of classical antibiotics.

## Materials and methods

A dip-coating method using a suspended liquid developed in our group by Hosseiny et al.^36^ was applied for coating PDS-II sutures (Ethicon Inc., Cincinnati, OH, US) with a variety of nanoparticles. PDS II are commercial synthetic absorbable monofilament sutures made from polyester (p-dioxanone), currently used in hospital on soft tissue^45^. Briefly, different aqueous solutions containing the specific metal nanoparticles (ZnO, TiO_2_, Fe_2_O_3_, Cu, Cu_2_O and MgO) (Sigma-Aldrich, Oakville, ON, Canada) was prepared by adding 0.1 M Na_2_SO_4_ (Sigma Aldrich, Oakville, ON, Canada), 30 mM of ascorbic acid (Sigma Aldrich, Oakville, ON, Canada) and the desired nanoparticle in 25 ml of ultrapure water (Milli‐Q, Merck, Darmstadt, Germany). For each nanoparticle, a specific concentration was added following the protocol. The resultant solution was mechanically agitated using an Electrode Rotator (Model 616A, Princeton Applied Research, Oak Ridge, TN, USA) equipped with a Teflon™ circular rod for several hours, allowing a film of nanoparticles at the liquid surface. All solutions were agitated at 1500 RPM at room temperature. The size of nanoparticles, concentrations and time of agitation are showed in Table 3. PDS-II sutures were coated while continually agitating of the liquid. Sutures were cut in small pieces (5 cm and 15 cm) and reserved. The coating was applied by dipping sutures (individually) in the suspending liquid after the determined time. Sutures were dipped 5 times using a tweezer to hold them, with 10 min interval between each coating.

**Table 3:** Particle size (nm), concentration (g/ml) and time of agitation (h) for different nanoparticles applied to the PDS-II sutures coating.

Nanoparticle	Size (nm)	Concentration (g/ml)	Time of agitation (h)
ZnO	25	0.030	4
TiO	50	0.006	4
Fe_2_O_3_	50	0.050	4
Cu	25	0.040	6
CuO	50	0.040	6
MgO	50	0.030	4

A thin layer of silk fibroin was applied after the last dip-coating process aiming to avoid detachment of nanoparticles. Silk fibroin solution was prepared according to the protocol by Rockwood et al.^46^. Briefly, *Bombyx mori* silkworm cocoons (donated by University of Würzburg, Germany) were boiled for 30 min in a solution of 0.02 M Na_2_CO_3_ (Sigma‐Aldrich, Oakville, ON, Canada) to remove sericin. The extracted silk fibroin fibers were rinsed in ultrapure water (Milli‐Q, Merck, Darmstadt, Germany) and set to dry for 24 h inside a fume hood at room temperature. Dried silk fibroin was dissolved in a 9.3 M LiBr (Sigma‐Aldrich, Oakville, ON, Canada) aqueous solution at 60 °C for 4 h. The solution was dialyzed against ultrapure water using dialysis tube (MEMBRA‐CEL dialysis tubing, MWCO 3500, Fisher Scientific, Scotia Court, ON, Canada) at room temperature for 48 h. The obtained silk fibroin solution (6.5% wt.) was purified using centrifuge (Centra MP4/MP4R, Fisher Scientific, Scotia Court, ON, Canada) for 20 min, 4500 rpm, 4 °C, to remove impurities. The precoated PDS-II sutures were dipped one time in the resultant silk fibroin solution and dried at room temperature. The resultant PDS-II coated sutures were sterilized using 70% Ethanol (Sigma Aldrich, Oakville, ON, Canada) at UV-light overnight and reserved in a closed recipient to avoid any contamination.

The morphology and homogeneity of particles were investigated by Scanning Electron Microscopy (SEM, Inspect F50, FEI Company, Hillsboro, OR, USA) in uncoated and coated PDS-II sutures. The diameter for uncoated and coated sutures was determined by measuring 3 different points along the length using ImageJ software. A skin model in rat cadavers was applied in order to investigate the attachment of the nanoparticles by dip-coating process. Briefly, the back area of cadavers was shaved before skin test performance. Uncoated and coated PDS-II sutures (5 cm length) were passed through the rat skin and further analyzed using SEM. The fresh rat cadavers were obtained from McGill animal facilities localized at Montreal General Hospital (Montreal, QC, Canada). The tensile strength of the coated and uncoated PDS-II sutures was tested using a loop model. Briefly, 15 cm sutures were set in a 7 cm circumference loop (Supplementary data Fig. S1), using an Omegadyne model LCCD-100 (Frystatska, Czech Republic). 100 lb/450 N capacity (0.03% FS linearity, 0.02% FS hysteresis, 0.01% FS repeatability). All sutures were knotted using a reef square knot to secure the sutures attachment. The sutures were held in the support and a separation rate of 0.7 mm/s was applied. The values for break load and extension at break load were reported. The tensile modulus was calculated from the initial slope of the stress–strain curve using linear regression method. Five samples for each different coating applied were tested. In vitro degradation behavior of coated and uncoated PDS-II sutures was investigated in the presence of protease XIV from Streptomyces griseus (Sigma Aldrich, Oakville, ON, Canada) adapting Franco et al. method^39^. First, all sutures (1 cm length) were weighted and recorded as ${M}_{i}$. Pre-weighed sutures were immersed in 2 mL of phosphate-buffered saline (PBS-Sigma Aldrich, Oakville, ON, Canada) solution containing 1 U/mL protease and kept for 0, 1, 3, 5 and 7 days incubated at 37 °C. The enzymatic solution was changed every 2 days. After each degradation period, samples were rinsed using ultrapure water, dried overnight at 37 °C and, finally, weighed and recorded as ${M}_{f}$. The percentage of weight-loss was determined according to Eq. (1)^39^. Three samples per different coating applied were tested.

1 $$\% Weight \,  loss=\frac{{M}_{i}-{M}_{f}}{{M}_{i}} x 100\%$$

The amount of ROS produced from uncoated and coated PDS-II sutures was investigated following Vieira et al. protocol^25^ with an intracellular ROS‐indicator, DCFH‐DA. This quantitative assay is based on the oxidation of the nonfluorescent DCFH to highly fluorescent DCF by ROS^26,27^. Briefly, uncoated and coated PDS-II sutures (1 cm length) were added (individually) in each well of a 12 well plate filled with 1 mL of PBS. The experiment was performed at 1, 3, 5 and 7 days, keeping the samples incubated at 37 °C. After each time point, 0.5 mL of each well was added to 12.5 µL of 25 × 10^−6^ M DCFH‐DA (Sigma‐Aldrich, Oakville, ON, Canada) ethanol solution and incubate for 10 min protected from light exposure with aluminum foils, at room temperature. The final mixture was analyzed with a fluorescence plate reader (Infinite M200 Pro TECAN, Tecan Trading AG, Switzerland) at excitation and emission wavelength of 488 and 525 nm, respectively. Three samples per different coating applied were tested.

In vitro cytotoxicity tests were performed. Standard mammalian cell culture techniques were employed to maintain Chinese hamster ovary cells (CHO) (ATCC®: The Global Bioresource Center, Manassas, VA, US) in Gibco alpha-MEM supplemented with 10% Fetal Bovine Serum (FBS) as well as 1% penicillin–streptomycin (Thermofishcer Scientific™, Scotia Court, ON, Canada) and 10 mM HEPES buffer in an incubator at 37 ˚C injected with 5% CO_2_. Metallic nanoparticle coated PDS II sutures were tested against CHO mammalian cell cultures to examine cytotoxicity. CHO cells were seeded into 10 mL of medium and maintained as a monolayer in T75 corning flasks until 85% confluent. The cells were trypsinized using Trypsinogen and counted using Trypan Blue Staining to ensure appropriate confluence prior to plating. Approximately 4 × 10^5 cells were counted for each T75 flask. Cells were plated in a 24-well plate with 1 mL of culture placed into each well. Suture samples were allowed to sterilize overnight using 70% ethanol and UV-light prior to insertion into the wells. For each time point (1, 3, 5, 7 days), the sutures were removed from the wells with care taken not to touch the base. The cell survival rate was observed through Hoechst staining using FluoReporter® (Thermofishcer Scientific™, Scotia Court, ON, Canada) Blue Fluometric dsDNA quantitation kit (F-2962) following FluoReporter® protocol guidelines. A DNA quantitation standard curve was generated using standardized CHO DNA samples. Once a DNA quantitation standard curve was established, the experimental samples were prepared by allowing cells to adhere to a microplate. Once adhered, the plates were emptied of medium by overturning the plates onto paper towel. 100 μL of distilled water was added to each well and allowed to incubate at 37 °C for 1 h. The microplate was then frozen at -80 °C to lyse cells then thawed at room temperature. 100 μL of Hoechst stain in TNE buffer was added to each well. The fluorescence was then measured using excitation and emission filters centered at 360 nm and 460 nm, respectively. The data were plugged in to the standard curve equation to express the data in percentages of live cells. Pristine suture was used as negative control for normalization.

In vitro bactericidal activity was assessed. Metallic nanoparticle sutures were tested against *Pseudomonas aeruginosa* (ATCC® 15,442™: The Global Bioresource Center, Manassas, VA, US) and *Staphylococcus aureus* (ATCC® 29,213™: The Global Bioresource Center, Manassas, VA, US) cultures to examine bacterial toxicity. All bacterial samples were prepared through overnight cultures in Luria broth (LB) medium 17.5 h before suture application. The bacterial samples were aliquoted such that there was one sample for each time point (1, 3, 5, and 7 days) per metallic nanoparticle treatment, including an uncoated suture control and a killed bacteria positive control. Each 5 mL sample of either *P. aeruginosa* or *S. aureus* were prepared in 25 mL conical Falcon® tubes. After all samples were properly aliquoted, a 4 cm segment of suture was applied to the sample. Treated samples were kept at 4 °C for the duration of the experiment and until the samples reach their respective timepoints. Once a timepoint had been reached, the corresponding set of samples for each treatment were prepared for a live/dead bacterial assay to assess the degree of dead bacteria. The samples were first pelleted by centrifugation at 10,000*g* in a microcentrifuge for 10 min then resuspended in 2 mL of PBS. Then 1 mL from each of the bacterial cell samples in PBS was diluted in 5 mL Abcam Bacterial Viability assay kit Wash Buffer. The samples were pelleted, re-suspended and diluted again before being stained using the LIVE/DEAD Abcam Bacterial Viability assay kit (ab189818). After staining, the samples were incubated in complete darkness for 1 h. After incubation, the samples were pipetted into a 96-well plate in triplicate then to assess fluorescence using excitation and emission filters centered at 490 nm and 536 nm, respectively and 525 nm and 617 respectively. The excitation and emission filters centered at 525 nm and 617 nm correspond to the dead cell stain. The excitation and emission filters centered at 490 nm and 536 nm correspond to the whole cell stain. Once fluorescence readings were acquired, the readings were used to produce a fraction representing the dead portion of the bacteria in the sample. All steps were taken from the LIVE/DEAD Abcam Bacterial Viability assay kit (ab189818, Waltham, MA, US) protocol.

### Statistical analysis

The data were analyzed using OriginPro (OriginLab Corporation, version 2018G) and GraphPad Prism 8.4.2. (Software Mackev 2020). The data were presented as mean ± SD. One‐way ANOVA tests were performed to evaluate statistical significance between uncoated and coated PDS-II sutures. Tukey tests were performed to analyze statistical significance between different coatings proposed at each time point. *P*‐values smaller than 0.05 were considered to express significant difference.

## Supplementary Information

Supplementary Information.
