# Supplementary material for: Engineering surgical stitches to prevent bacterial infection
Source: Sci Rep. 2022 Jan 17;12:834. doi: 10.1038/s41598-022-04925-5 (PMC8764053; doi:10.1038/s41598-022-04925-5)
Supplement: Supplementary file 1 — Supplementary Information. [file 41598_2022_4925_MOESM1_ESM.docx]

**Engineering surgical stitches to prevent bacterial infection**

*Daniela Vieira, Samuel N. Angel, Yazan Honjol, Maude Masse, Samantha Gruenheid, Edward J. Harvey, Geraldine Merle*

**Supplementary data**


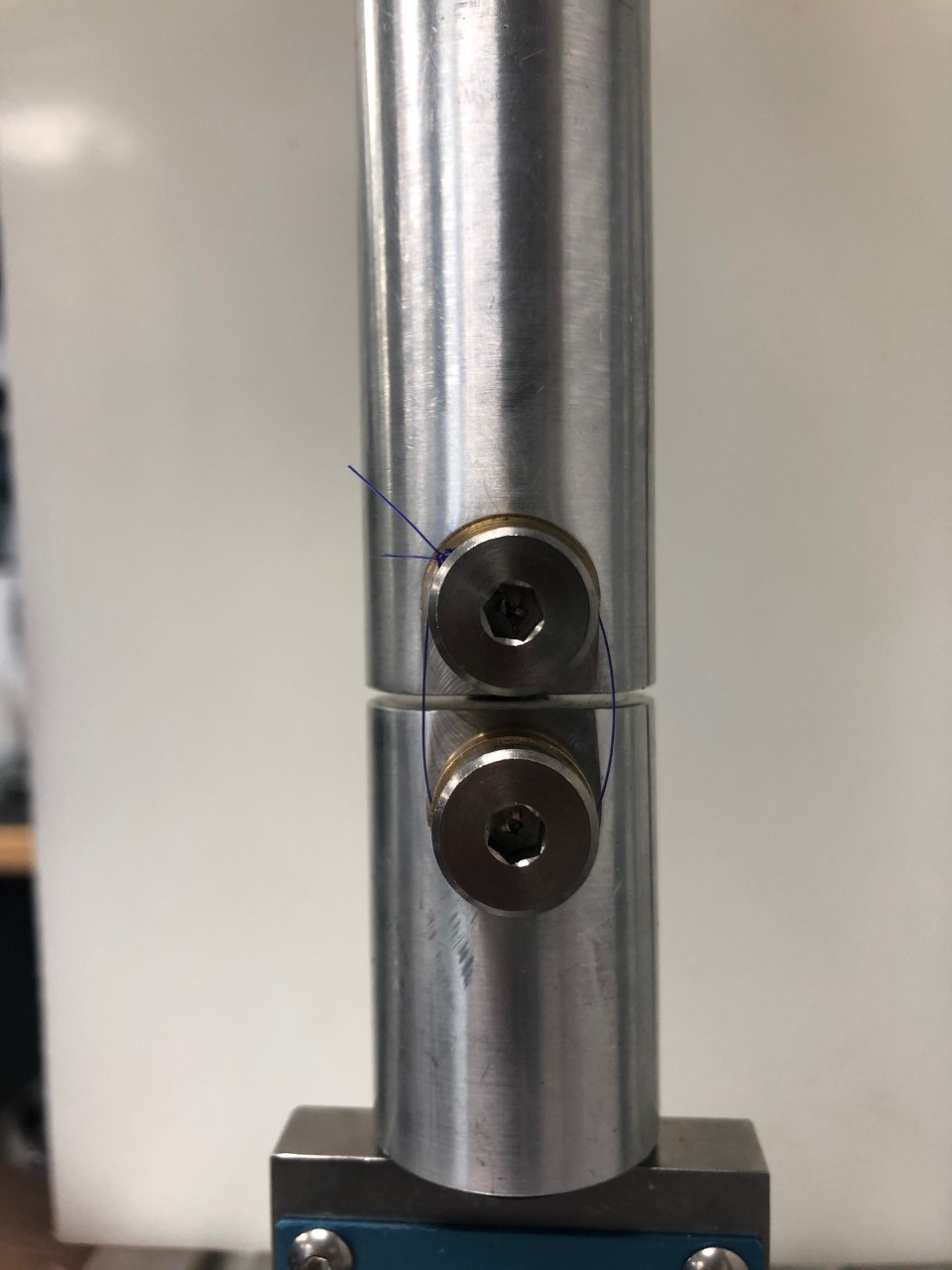


Figure S1 - The loop model applied in the mechanical tensile test. Suture loop (70 mm diameter) was held in the support and a separation rate of 0.7 mm/sec was applied.

**Table S1 – Uncoated and coated sutures diameters**

| **Suture coating** | **Diameter (µm)** | **Standard deviation** |
| --- | --- | --- |
| Control (PDS II)^(a)^ | 224.15 | 0.81 |
| TiO^(a)^ | 236.88 | 1.77 |
| ZnO^(a)^ | 227.89 | 0.43 |
| CuO^(a)^ | 241.17 | 1.59 |
| Cu^(a)^ | 243.34 | 1.16 |
| Fe2O3^(a)^ | 233.40 | 0.84 |
| MgO^(a)^ | 231.88 | 0.54 |

^(a)^ANOVA test p<0.05 – significant difference

**Table S2 - Stress and strain values for suture conditions. All values represent stress and strain exerted on the suture at the moment of fracture indicating the maximum stress and strain.**

| **Suture** | **Strain (%)** | **Stress (GPa)** |
| --- | --- | --- |
| PDS II | 12.7 ± 1.73 | 1.61 ± 0.07 |
| Silk | 11.34 ± 2.65 | 1.44 ± 0.54 |
| TiO | 7.52 ± 0.57 | 1.72 ± 0.38 |
| ZnO | ^(a)^5.31 ± 1.84 | 1.71 ± 0.06 |
| CuO | 10.25 ± 2.26 | 1.86 ± 0.07 |
| Cu | ^(a)^5.49 ± 1.28 | 1.78 ± 0.12 |
| Fe2O3 | 13.11 ± 1.24 | 2.02 ± 0.08 |
| MgO | ^(a)^6.55 ± 1.14 | 2.06 ± 0.09 |

^(a)^ANOVA test p<0.05 – significant difference, n=5


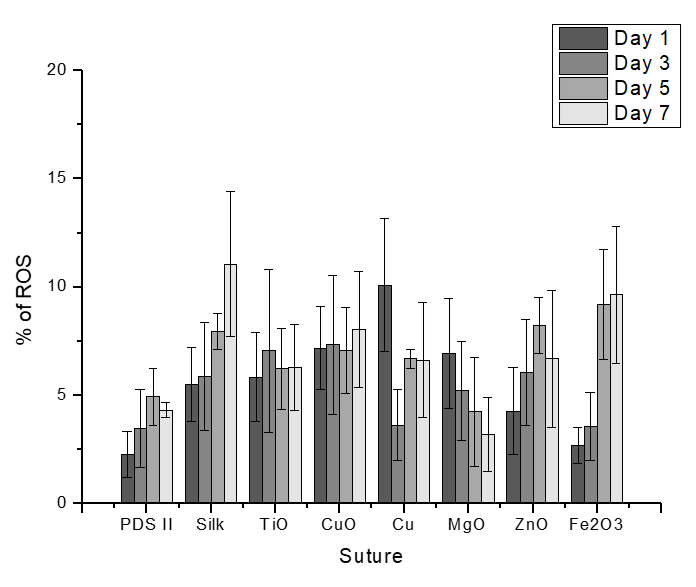


**Figure S2.** ROS production represented by % of ROS in the aqueous suture testing environment at each timepoint (1, 3, 5, 7 days) (n= 3). ANOVA and Tukey tests were performed with the weight differences in coated sutures against weight differences in the uncoated PDS II samples.
